# Second Generation Drug-Eluting Stent Implantation versus Coronary Artery Bypass Grafting in the Treatment of Young Patients with Left Main and/or Multivessel Coronary Disease

**DOI:** 10.1155/2020/6736704

**Published:** 2020-04-20

**Authors:** Xue Chen, Xuehui Zhang, Yunfeng Yan, Xin Zhao, Maoxiao Nie, Tingting Feng, Zhe Liang, Quanming Zhao

**Affiliations:** Beijing Anzhen Hospital, Capital Medical University, Beijing Institute of Heart, Lung and Blood Vessel Diseases, The Key Laboratory of Remodelling-related Cardiovascular Diseases, Department of Cardiology, 2 Anzhen Road, Chaoyang District, Beijing 100029, China

## Abstract

**Background:**

Many studies have compared the outcomes of coronary artery bypass graft (CABG) and percutaneous coronary intervention (PCI) for complex coronary artery disease (CAD). However, no trials have focused on young patients (<45 years) with complex CAD. We conducted a retrospective evaluation to compare the outcomes of a second-generation drug-eluting stent (DES) and CABG in young patients with LM or three-vessel disease.

**Methods:**

In young patients with complex CAD who underwent PCI or CABG, a Kaplan-Meier analysis and Cox regression before and after propensity score matching were used to compare major adverse cardiac and cerebrovascular events (MACCE), including myocardial infarction (MI), stroke, death, and repeat revascularization.

**Results:**

During follow-up, MACCE occurred in 20.5% of patients in the PCI group and 8.6% of patients in the CABG group (hazard ratio (HR): 3.263, 95% confidence interval (CI): 1.379 to 7.722, *p*=0.007). Repeat revascularization occurred more frequently in the PCI group (18.9% vs. 3.7%, respectively, HR: 6.968, 95% CI: 2.036 to 23.842, *p*=0.002). There were no significant differences in the other endpoints. After propensity score matching, no conclusions were modified.

**Conclusions:**

In young patients with LM or three-vessel disease, PCI showed a higher incidence of MACCE, which was mainly driven by repeat revascularization. However, this did not translate into hard endpoint differences. Therefore, PCI is an alternative treatment to CABG in young patients with complex CAD.

## 1. Background

Left main (LM) disease and three-vessel disease are types of complex coronary artery disease (CAD), the treatment of which is more difficult. Coronary artery bypass graft (CABG), as an effective treatment for CAD, has been introduced for more than 50 years, and it is currently the preferred modality for treating complex CAD [1, 2]. However, over the last twenty years, there have been significant advances in percutaneous coronary intervention (PCI) from the era of balloon angioplasty and subsequent bare-metal stents to drug-eluting stents (DESs) [3]. With improving technology and techniques for PCI, such as adjunctive antithrombotic drugs, periprocedural management, and the experience of interventional cardiologists, research has focused increasingly on more complex diseases, such as LM disease and multivessel disease.

The latest European Society of Cardiology (ESC) and European Association for Cardio-Thoracic Surgery (EACTS) guidelines [4] recommend CABG (class I, level A) for complex CAD, including both LM disease and three-vessel disease regardless of the anatomic complexities of coronary arteries. However, PCI is an alternative in the case of LM disease and three-vessel disease if the SYNTAX score is ≤ 22 (class I, level A); if the SYNTAX is > 22, PCI would be inferior for LM disease and three-vessel disease (class II or III, level A or B). Even so, along with the rapidly progressing technology, an increasing number of patients and cardiologists prefer PCI over CABG according to many trials. Several trials have reported that PCI is noninferior to CABG in patients with LM disease [5] or multivessel disease [6]. On the other hand, many trials have suggested that CABG might provide better clinical outcomes than PCI [7].

In view of the younger characteristics of CAD patients and the likelihood of graft failure and considering that 62% of patients will have recurrent ischemia by 15 years after operation [8], it is essential to consider the risk/benefit ratio of PCI and CABG for LM and three-vessel disease by weighing the procedural invasiveness and the associated short-term complications against long-term event rates of death, myocardial infarction (MI), stroke, repeat revascularization, and improvements in health-related quality of life [9]. Therefore, we hypothesize that PCI should be performed at an early age if possible to alleviate symptoms to provide another choice for advanced illness. We conducted a retrospective evaluation to compare real-world outcomes between CABG and PCI using second-generation DESs in young patients with LM disease or three-vessel disease.

## 2. Methods

This was a single-center retrospective study comparing PCI with second-generation DES and CABG in young patients with LM and three-vessel disease. We performed a review of all young patients who underwent PCI or CABG at Beijing Anzhen Hospital from January 2015 to December 2016. We screened these patients and enrolled them if they had LM and/or three-vessel disease, and they underwent PCI with a second-generation DES or CABG. The patients who had previously undergone PCI were also included in our study. Patients were excluded if (1) they did not suffer from LM or three-vessel disease; (2) they had acute myocardial infarction (MI), either ST-segment elevation or non-ST-segment elevation; (3) they underwent concomitant valvular or aortic surgery; (4) they had previous CABG surgery; and (5) they were unable to receive both procedures or refused to.

In our trial, the revascularization strategy was determined by the physicians' and/or patients' preferences based on hemodynamic conditions, anatomic factors, vessel size, the presence of comorbidities, and the quality of arterial and/or venous conduit graft fit.

All PCI procedures were performed according to current standard interventional guidelines. Antiplatelet therapy and periprocedural anticoagulation followed standard regimens. All patients received 300 mg of loading dose aspirin and/or clopidogrel (or 180 mg ticagrelor) before the procedure. After PCI, all patients were recommended 100 mg/day aspirin indefinitely and 75 mg/day clopidogrel or ticagrelor 90 mg twice daily for at least 1 year. There was no restriction for the second-generation DESs.

The bypass graft revascularization was performed with standard bypass techniques. A normal midline sternotomy incision was used to expose the heart, and both on-pump and off-pump surgeries were performed at the preference of the surgeon. The internal thoracic artery was preferred for bypass of the left anterior descending artery. After CABG, medications were given according to the guidelines or the preference of the surgeon.

Patient data on demographics, comorbid conditions, laboratory echocardiography, procedures, and so on were collected via chart review. The ethics committee at our hospital (Beijing Anzhen Hospital) reviewed our study protocol and approved the use of clinical data for the study (No : 2018020X). Because of the retrospective nature of our trial, the need for informed consent was waived. Follow-up and information on the clinical status were obtained from clinical visits and telephone interviews.

The primary endpoint in our trial was major adverse cardiac or cerebrovascular events (MACCE), a composite of all-cause death, stroke, myocardial infarction (MI), and repeated revascularization. The secondary endpoints were the individual occurrence of all-cause death, stroke, MI, and repeat revascularization. Deaths were considered cardiac unless unequivocally noncardiac. Stroke was defined as a focal neurological deficit of central origin lasting >24 h as confirmed by a neurologist and computed tomography or magnetic resonance imaging. MI was defined as a creatine kinase-MB level >50 ng/ml or the appearance of new Q-waves or ST-segment elevation >2 mm on the electrocardiogram which were confirmed by a veteran cardiologist. Repeated revascularization in our trial was any revascularization that was performed on any vessel by PCI or CABG.

Young patients were defined as <45 years old according the World Health Organization (WHO). Lesion of each vessel, including its main branches (diameter ≥1.5 mm), was defined as ≥50% stenosis. Multivessel disease or three-vessel disease was ≥50% stenosis in all three epicardial coronary arteries, consisting of the left anterior descending artery (LAD), left circumflex artery (LCX), and right coronary artery (RCA) or their main branches. Other definitions, such as hypertension (HT) and diabetes mellitus, were based on the international guidelines or medication management. The baseline and outcome data for individual patients were pooled. Continuous variables are expressed as the mean ± standard deviation (SD) and were compared using Student's *t*-test. Categorical variables are presented as frequencies and proportions, and comparisons were performed using the chi-square test or Fisher's exact text, as appropriate.

The rate of cumulative events and incidence curves for clinical outcome were estimated using the Kaplan-Meier method. A Cox proportional hazard regression was performed to determine independent predictors of MACCE, MI, stroke, death, and repeat revascularization in univariate and multivariate analyses. The analyses were used to determine the noninferiority of PCI and identify the adjusted hazards. The adjusted covariates included operation strategy (PCI/CABG), age, sex (male/female), smoking (yes/no), alcohol (yes/no), body mass index (BMI), HT (yes/no), DM (yes/no), hypercholesterolemia (HC, yes/no), family history (yes/no), creatinine clearance rate (CCR), uric acid (UA), C-reactive protein (CRP), blood group (A, B, AB or O), left ventricular ejection fraction (LVEF), prior MI (yes/no), prior heart failure (HF, yes/no), prior stroke (yes/no), prior stent (yes/no), collateral circulation (yes/no), lesion type (LM or three-vessel disease), and Gensini score. Multivariable predictors of outcomes were identified using forward stepwise selection with a significance level of <0.05 for the entry and exit criteria. Finally, the significant factors and several clinically important factors entered subsequent analysis. The results are reported as the hazard ratio (HR) and 95% confidence interval (CI).

To reduce selection bias and any other related, potentially confounding factors, we performed a baseline characteristic adjustment for patients using the propensity score. The propensity scores were estimated using a multiple logistic regression model. A full nonparsimonious model was developed that included all variables listed in Tables 1 and 2. The patients who underwent PCI and the patients who underwent CABG were then matched at a 1 : 1 ratio by propensity score using a nearest-neighbor matching algorithm with a caliper of 0.01. The absolute standardized differences of variables included in the calculation of propensity score were compared before and after propensity score matching. Standardized differences <10% for these included variables indicated a relatively better balance. The baseline characteristics and outcomes between the two propensity score-matched subsets were recompared.

Subgroup analyses, including sex, HT, DM, prior MI, prior HF, lesion type, and Gensini score, were run using the Cox proportional hazard model. The variables included in the model were significant and were proven in the model design or clinically relevant. Texts for interaction were performed to assess the heterogeneity of the treatment effect among subgroups.

A two-sided *p* value of less than 0.05 was considered to indicate significance. All statistical analyses were performed using SPSS software version 20.0 (IBM Corporation) or Stata version 14.0 (StataCorp, College Station, TX, USA).

## 3. Results

**Figure 1:**
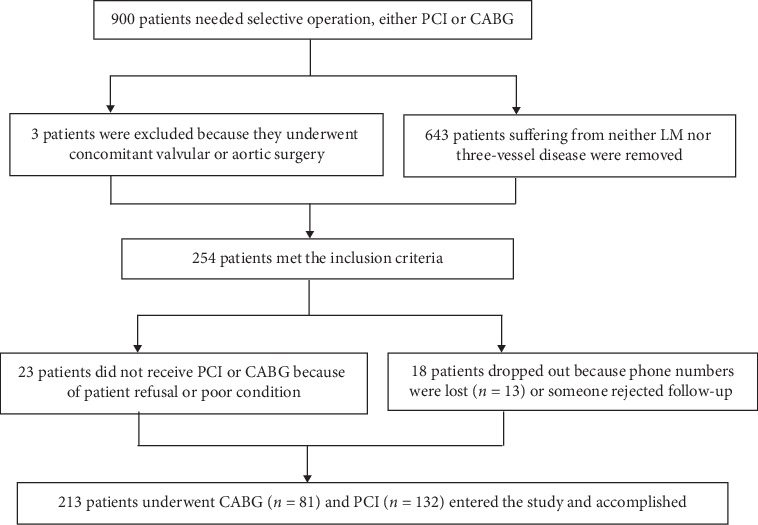
Flow chart of enrolled patients. PCI: percutaneous coronary intervention; CABG: coronary artery bypass grafting; STEMI : ST-segment-elevation myocardial infarction; NSTEMI: non-ST-segment-elevation myocardial infarction; LM disease: left main coronary artery disease.

Between January 2015 and December 2016, a total of 900 patients underwent PCI or CABG at our hospital. However, 687 patients were removed from our trial by the inclusion and exclusion criteria, leaving 213 patients who entered the study, including 81 CABG and 132 PCI with second-generation DESs (Figure 1).

The baseline demographic characteristics are listed in Table 1. In the overall population, the incidence of smoking, hyperlipidemia, and several physical and biochemical indexes such as BMI, CCR, and UA were higher in the patients in the PCI group, whereas the Gensini score (69.80 ± 35.10 vs. 86.64 ± 36.77, *p*=0.001) showed the opposite difference. The antiplatelet drug P_2_Y_12_ inhibitor was significantly more common in patients in the PCI group than in patients in the CABG group. There were no differences between treatment groups in terms of age, sex, HT, DM, LVEF, prior MI, prior HF, lesion type, or other factors.

**Figure 2:**
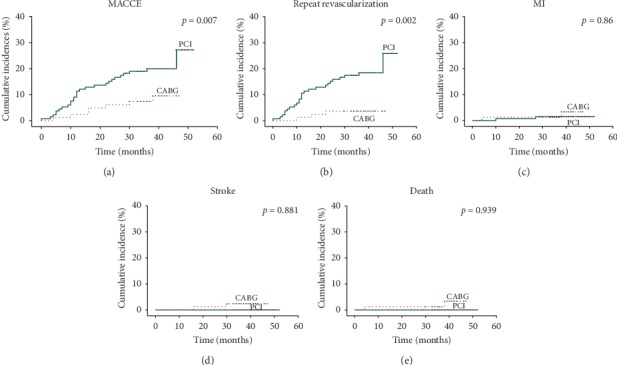
Kaplan-Meier cumulative event curves of MACCE and secondary endpoints before propensity score matching. The adjusted risk of PCI relative to CABG is shown. PCI: percutaneous coronary intervention; CABG: coronary artery bypass graft; MI: myocardial infarction.

The median duration of follow-up among all patients was 38 months (interquartile range: 36 to 41 months). The cumulative incidences of clinical outcomes of all patients are described in Table 2 and Figure 2. Between the follow-ups, the incidence of MACCE was 20.5% in the PCI group and 8.6% in the CABG group (unadjusted HR: 2.508, 95% CI: 1.091 to 5.762, *p*=0.03; adjusted HR: 3.263, 95% CI: 1.379 to 7.722, *p*=0.007). The incidence of repeat revascularization was 18.9% in the PCI group and 3.7% in the CABG group (unadjusted HR: 5.435, 95% CI: 1.64 to 18.011, *p*=0.006; adjusted HR: 6.968, 95% CI: 2.036 to 23.842, *p*=0.002). There were no significant differences in other endpoints, such as MI, stroke or death, before or after adjusting for multiple variables and clinical background (1.5% vs. 2.5%, *p*=0.86; 0 vs. 2.5%, *p*=0.881; 0 vs. 2.5%, *p*=0.939 after adjustment, respectively).

**Figure 3:**
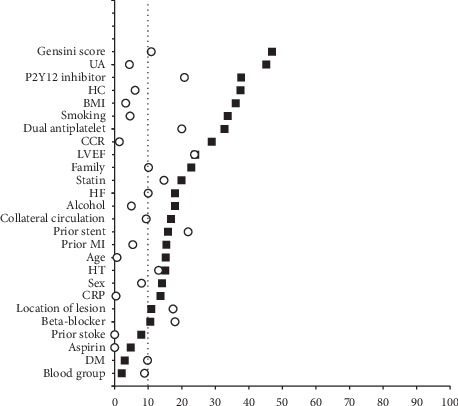
Standardized differences before and after propensity score matching. An absolute standardized difference of less than 10% indicates a good match. BMI: body mass index; CABG: coronary artery bypass grafting; CCR: creatinine clearance rate; CRP:C-reactive protein; DES: drug-eluting stent; HF: heart failure; LM: left main; LVEF: left ventricular ejection fraction; MI: myocardial infarction; PCI: percutaneous coronary intervention; UA: uric acid.

After propensity score matching was performed, there were 46 matched pairs of patients in the two groups. The baseline characteristics of the patients after propensity score matching with no significant differences were found between the two groups and more variables showing standardized differences less than 10%; an additional figure file shows this in more detail (see Figure 3). Compared with the CABG group, the PCI group had a higher prevalence of MACCE (32.6% vs. 10.9%, HR: 4.496, 95% CI: 1.592 to 12.695, *p*=0.005) and repeat revascularization (30.4% vs. 4.3%, HR: 11.6, 95% CI: 2.449 to 55.51, *p*=0.002) after adjustment. However, as in the overall population, there were no significant differences in the incidence of MI (2.2% vs. 2.2%, *p* = 0.55), stroke (0 vs. 4.3%, *p*=0.658), or death (0 vs. 2.2%, *p*=0.876).

**Figure 4:**
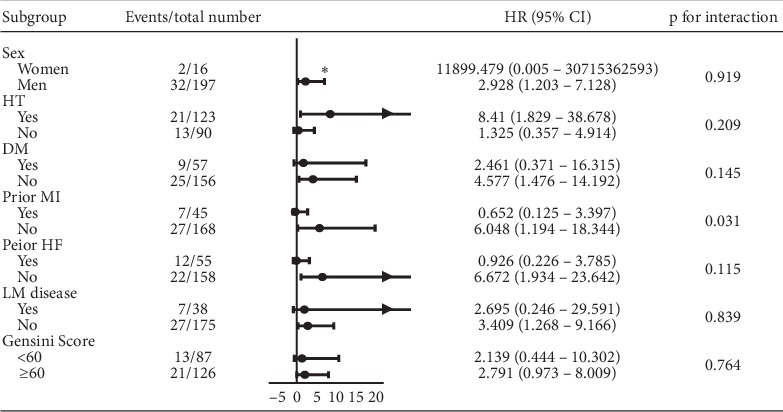
Subgroup analysis based on sex, HT, DM, prior MI, prior HF, LM disease, and Gensini score. HT: hypertension; DM: diabetes mellitus; MI: myocardial infarction; HF: heart failure; LM disease: left main coronary artery disease, which includes LM coronary artery disease in isolation and LM coronary artery disease with multivessel disease (three-vessel disease). ^∗^Because the data point is outside the axis limits.

Subgroup analyses were performed based on important baseline characteristics. There were no significant interactions between any treatment effects of PCI versus CABG in the rate of MACCE except for the prior MI (*p* for interaction = 0.031). PCI was associated with an increased risk of MACCE in other subgroups. However, the patients who had ever suffered MI experienced a lower rate of MACCE in the PCI group than in the CABG group (HR: 0.652, 95% CI: 0.125 to 3.397) (see Figure 4). In the multivariate Cox regression analysis, age (HR: 1.147, 95% CI: 1.004 to 1.312, *p*=0.044), CRP (HR: 1.011, 95% CI: 1.005 to 1.016, *p* ≤ 0.001), Gensini score (HR: 1.013, 95% CI: 1.004 to 1.022, *p*=0.006), and operation strategy (HR: 3.263, 95% CI: 1.379 to 7.722, *p*=0.007) were found to be predictors of MACCE.

The predictors of repeat revascularization were CRP (HR: 1.011, 95% CI: 1.005 to 1.016, *p* ≤ 0.001), Gensini score (HR: 1.012, 95% CI: 1.002 to 1.022, *p*=0.018), and operation strategy (HR: 6.968, 95% CI: 2.036 to 23.842, *p*=0.002). There were no significant predictors of other endpoints.

## 4. Discussion

In this retrospective study, CABG was shown to be superior to PCI with second-generation DESs in young patients with LM and/or three-vessel disease in terms of the incidence of MACCE, which was driven mainly by repeat revascularization. There were no significant differences in the hard endpoints death, MI, and stroke in line with the outcomes of the recent EXCEL trial [5]. A recent meta-analysis which demonstrated no difference between PCI and CABG for the treatment of LM disease in the composite endpoint of death, stroke, and MI while the rate of repeat revascularization was substantially higher in the PCI group also partly supports our results [10]. After adjustment by propensity score matching to minimize selection bias, the conclusion was the same as in the overall population. Although this study was limited by its observational design, this is the first report specifically addressing the issue of LM and three-vessel disease in young patients, and it evaluated the potential noninferiority of PCI over CABG. Therefore, our results would be helpful when making a clinical decision in real-world practice, especially for young CAD patients.

It is good that young patients have better baseline characteristics along with fewer and milder complications, so it is essential to consider the broad indications and long-term prognosis. In our study, there were no obvious differences between the two groups in terms of baseline characteristics, except that the CABG group had more complex coronary anatomy and lower usage of dual antiplatelets. However, the PCI group showed a higher incidence of MACCE and repeat revascularization.

As in some previous studies, we found that CABG was better than PCI for the composite endpoint of MACCE and repeat revascularization both in LM [11] and three-vessel disease [12]. One of the reasons is that after PCI progressive atherosclerosis can lead to new, severe stenosis and plaque rupture that may cause ischemia and repeat revascularization, and CABG offers better protection by bypassing a large proportion of obstructive lesions or vulnerable plaques, minimizing the impact of progressive disease in the entire upstream proximal vessel [13]. Moreover, there was more incomplete revascularization in the PCI group that needed more than one intervention operation, whereas patients achieving complete revascularization showed similar outcomes between PCI and CABG [14]. In addition, more routine angiographic follow-up was performed to detect early in-stent restenosis in the patients treated with PCI rather than in those treated with CABG. Many patients with PCI receive repeat revascularization that is angiographically rather than clinically driven. Thus, the rate of repeat revascularization might be underestimated for the patients undergoing CABG. We must recognize that with the introduction of high-pressure deployment, the use of intravascular ultrasound and improved stent design, restenosis of drug-eluting stents has diminished over time [15].

Some studies show that the PCI group had higher rates of MI [16], whereas other studies [17] support our finding that there were similar rates of MI between the PCI and CABG groups. The main advantage of CABG might be the bypassing of long lesion segments by grafting, which protects, to a great extent, against target lesion MI and proximal de novo lesion MI [15]. The small population and short follow-up time may be two of the reasons that caused the absence of significant differences in MI rates.

While some other studies showed that CABG resulted in significantly higher rates of stroke than PCI for LM or multivessel disease [3], we found that the difference in rates was indistinctive. The mechanisms underlying the increased risk of stroke with CABG are likely multifactorial. First, CABG performed on-pump with cannulation and clamping of the aorta increases the rates of stroke, which may be reduced by an off-pump procedure [18]. Furthermore, stroke may be less common after PCI due to the routine use of dual antiplatelets after stent implantation. However, in the present study, the CABG group also had a higher usage of aspirin and clopidogrel or ticagrelor.

Partly different from Head's study [19], we and Park et al. shared the same outcome in terms of death to a certain extent [20]; i.e., there was no significant difference in the rate of death between the PCI and CABG groups. The low mortality after treatment in both groups showed that modern revascularization techniques and adjunctive therapy can lead to excellent survival in young patients with LM and three-vessel disease. These low incidences of MI, stroke, and death might be related to the young characteristics of the patients enrolled in our study.

In the subgroup analysis, we found that CABG might lead to higher rates of MACCE in patients who had previously suffered MI, whereas in the other patients, PCI caused more MACCE. No relevant studies support this discovery, so it will be important to conduct further studies to see if this finding is generalizable.

In contrast with previous reports involving multivessel or LM disease in part, we found that along with operation strategy the predictors of MACCE and repeat revascularization were age, CRP, and Gensini score. It is possible that the inflammation condition and coronary anatomy play an important role in the long-term curative effect, and this has been verified by other studies. Kosmoidou et al. [21] found that an elevated baseline CRP level was strongly associated with subsequent death, MI and stroke. Misumida et al. [22] determined that the SYNTAX score 2 was correlated with mortality. However, some predictors such as diabetes mellitus [23], heart failure [24], chronic renal failure [25], and so on, which have been proven to be related to MACCE in other studies, were not included in our study. From a clinical viewpoint, using the relevant variables that are considered potential predictors of MACCE in young patients with LM and three-vessel disease represents a first step in implementing further preventive measures and tailored therapies.

Considering the discussion above, patients in the PCI group with a second-generation DES had higher rates of repeat revascularization, which did not translate into a higher incidence of the hard endpoints of MI, stroke, and death. A recent meta-analysis and the PRECOMBAT study also support our results [11]. The young age of our patients could explain this result, but it is important to select appropriate operations for long-term survival. The relative benefits of CABG versus PCI with stents in terms of outcomes are highly debated, particularly with each advancement in stent design. Currently, the state-of-the-art stent is the second-generation DES, which is thinner and coated with a more biocompatible polymer and new “limus” drugs that allow less inflammation and a lower rate of restenosis than first-generation DESs [7]. PCI may also be preferred because of its improved early safety profile.

The internal mammary arteries have been widely used as conduits to the left anterior descending artery due to their long-term patency, and the advantage of CABG may be partially due to the completeness of revascularization [26]. Although high long-term patency of the internal mammary artery is expected, some vein graft degeneration can be expected beyond 5 years [15]. Multiple arterial grafting is associated with improved survival and a reduced requirement of reintervention compared with grafting of a single internal thoracic artery plus the saphenous vein [27]. In the current era, routine use of the right internal mammary artery has not been widely adopted despite its identical histological features to the left internal mammary artery due to technical difficulties and concerns about a potential increase in rates of bleeding and wound complication [28].

However, saphenous vein grafts typically present accelerated atherosclerosis resulting in a high rate of stenosis or occlusion of the graft, which contributes to higher morbidity and mortality [29]. In the case of graft failure, repeat revascularization after either PCI or CABG is necessary in a certain number of patients, if appropriate. Nevertheless, in addition to increased operation difficulty, the patients undergoing re-CABG have a 2- to 4-fold higher mortality than in this operation than in the first operation, whereas PCI in patients previously treated with CABG is associated with worse acute and long-term outcomes than native artery PCI [30].

Our study had some limitations. First, it was a nonrandomized, retrospective study, although we performed propensity score matching to minimize the potential selection bias and ascertainment bias. Second, the follow-up duration and number of enrolled patients might not be sufficient to evaluate the long-term outcomes of revascularization. Third, this was a single-center study that included only Chinese patients, and more ethnicities are required in further trials. Fourth, because the treatment choice was left to the physician or patients, selection bias was inevitable. Fifth, because of the nature of our retrospective trial, invasive functional evaluations, which are essential in complex CAD, especially in young multivessel patients, were not conducted on many patients. Moreover, some patients who underwent CABG had the angiography performed at outside hospitals rather than at our hospital, which affected our evaluation of the lesion. Finally, we substituted the prevalent SYNTAX score with the Gensini score to estimate the anatomic complexity due to practical considerations.

## 5. Conclusion

In our retrospective study evaluating PCI with second-generation DESs versus CABG in young patients with LM and/or three-vessel disease in the real world, the PCI group suffered higher rates of MACCE than the CABG group, which was driven by repeat revascularization. However, PCI did not translate into a higher incidence of hard endpoints, such as MI, stroke, and death. In our opinion, for these young patients, along with the technical development of second-generation DESs for PCI, higher use of IVUS and fractional flow reserve and new imaging techniques, such as OCT, may be an alternative treatment strategy to CABG for long-term prognosis. Of course, further research and longer follow-up durations are indispensable.

## Figures and Tables

**Table 1:** Baseline characteristic of overall patients.

	CABG *n* = 81	PCI with second-generation DES *n* = 132	*p*-value
Age (year)	42.15 ± 2.82	41.67 ± 3.49	0.295
Male	73 (90.1%）	124(93.9%)	0.305
Smoking (current or former)	46 (56.8%)	96 (72.7%)	0.017
Drinking (current or former)	17 (21%)	38 (28.8%)	0.207
Hypertension	43 (53.1%)	80 (60.6%)	0.281
Diabetes mellitus	21 (25.9%)	36 (27.3%)	0.829
Hyperlipidemia	7 (8.6%)	29 (22.0%)	0.012
BMI (kg/m^2^)	26.80 ± 3.18	28.05 ± 3.75	0.013
CCR (ml/min)	121..34 ± 27.41	130.00 ± 32.08	0.045
UA (*µ*mol/l)	347.56 ± 97.86	390.03 ± 90.05	0.001
CRP (mg/l)	2.59 ± 5.44	6.32 ± 38.02	0.383
Family history	16 (19.8%)	39 (29.5%)	0.113
Blood group			0.962
A	27 (33.3%)	43 (32.6%)	
B	22 (27.2%)	37 (28.0%)	
AB	9 (11.1%)	12 (9.1%)	
O	23 (28.4%)	40 (30.3%)	
LVEF (%)	59.95 ± 8.41	61.83 ± 7.25	0.085
Prior HF	17 (21.0%)	38 (28.8%)	0.207
Prior MI	14 (17.3%)	31 (23.5%)	0.282
Prior stroke	1 (1.2%)	3 (2.3%)	0.664
Prior stent	6 (7.4%)	16 (12.1%)	0.272
Collateral circulation	3 (3.7%)	10 (7.6%)	0.378
Lesion type			0.366
LM with/without three-vessel disease	12 (14.8%)	26 (19.7%)	
Three-vessel disease isolated	69 (85.2%)	106 (80.3%)	
Gensini score	86.64 ± 36.77	69.80 ± 35.10	0.001
<60	25 (30.9%)	62 (47.0%)	
≥60	56 (69.1%)	70 (53.0%)	
Discharge medication			
Aspirin	80 (98.8%)	131 (99.2%)	1.00
P_2_Y_12_ inhibitor	74 (91.4%)	131 (99.2%)	0.005
Dual antiplatelet	74 (91.4%)	130 (98.5%)	0.016
Statin	78 (96.3%)	131 (99.2%)	0.30
Beta blocker	70 (86.4%)	109 (82.6%)	0.457

BMI: body mass index; CABG: coronary artery bypass grafting; CCR: creatinine clearance rate; CRP:C-reactive protein; DES: drug-eluting stent; HF: heart failure; LM: left main; LVEF: left ventricular ejection fraction; MI: myocardial infarction; PCI: percutaneous coronary intervention; UA: uric acid.

**Table 2:** Clinical outcome of overall patients before adjustment by propensity score matching.

	PCI (*n* = 132)	CABG (*n* = 81)	Unadjusted HR (95% CI)	*p*-value	Adjusted HR (95% CI)	*p*-value
MACCE	27 (20.5%)	7 (8.6%)	2.508 (1.091, 5.762)	0.030	3.263 (1.379, 7.722)	0.007
MI	2 (1.5%)	2 (2.5%)	0.601 (0.085, 4.268)	0.611	1.222 (0.132, 11.271)	0.860
Stroke	0	2 (2.5%)	0.008 (0.00, 1535.874)	0.435	0 (0.00, 1.490E + 048)	0.881
Death	0	2 (2.5%)	0.008 (0.00, 1423.967)	0.436	0 (0.00, 7.074E + 143)	0.939
Repeat revascularization	25 (18.9%)	3 (3.7%)	5.435 (1.640, 18.011)	0.006	6.968 (2.036, 23.842)	0.002

CABG: coronary artery bypass grafting; CI: confidence interval; HR: hazard ratio; MACCE: major adverse cardiovascular or cerebrovascular events which is the composite of all-cause death, stroke, MI or repeat revascularization; MI: myocardial infarction; PCI: percutaneous coronary intervention.

## Data Availability

The data used to support the findings of this study are available from the corresponding author upon request.
